# The effectiveness of an intervention using LAWE (loveliness and well-being of employees) app to improve the employee’s mental well-being

**DOI:** 10.34172/hpp.42892

**Published:** 2024-10-31

**Authors:** Mohammad Ali Morowatisharifabad, Abedin Iranpour, Mohammadreza Rajabalipour

**Affiliations:** ^1^Department of Health Education and Promotion, School of Public Health, Shahid Sadoughi University of Medical Sciences, Yazd, Iran; ^2^Department of Health Education and Promotion, School of Public Health, HIV/STI Surveillance Research Center, Institute for Futures Studies in Health, Kerman University of Medical Sciences, Kerman, Iran

**Keywords:** Employees, Mental health, Southeastern Iran, The LAWE app, Well-being, Workplace

## Abstract

**Background:**

Organizations are increasingly seeking comprehensive approaches to improve employee mental well-being (EMW). This study aims to evaluate the effectiveness of interventions delivered through the "Loveliness and Well-being of Employees" (LAWE) app, focusing on five key predictors: "Keep Learning," "Connect," "Take Notice," "Be Active," and "Given".

**Methods:**

A quasi-experimental intervention was conducted with 110 participants (53 in the intervention group and 57 in the non-intervention group) from the Kerman University of Medical Sciences in Southeastern Iran. Participants were recruited from a census of all employees at four schools. The intervention involved an Android-based app designed to enhance EMW through pre-designed tasks. Six standard tools were used to assess EMW and its predictors. Data analysis was performed using IBM SPSS Statistics 28, employing Independent Samples T-Test, paired-samples t-test, one-way ANOVA, Spearman correlation coefficient and analysis of covariance (ANCOVA).

**Results:**

The intervention group showed a significant increase in mean EMW score, from 52.26±8.61 to 60.01±6.95 (*P*<0.01). Based on ANCOVA analysis, the post-intervention mean score was significantly higher in the intervention group compared to the non-intervention group by 0.219 points (*P*<0.01). The mean score of all predictors, except "Take Notice" (*P*=0.17), also significantly improved in the intervention group. Additionally, post-intervention comparison between the two groups revealed significant differences in mean EMW score (*P*<0.01), as well as in the mean scores for "Keep Learning" (*P*<0.05), "Given" (*P*<0.01), and "Connect" (*P*<0.01).

**Conclusion:**

The LAWE app provides a desirable and comprehensive platform for improving the EMW in organizations. It significantly improves most of the key predictors of EMW, making it a valuable resource for organizations aiming to promote employee well-being.

## Introduction

 The employee’s mental well-being (EMW) is a crucial factor that any organization should consider. This shift is highlighting the correlation between EMW and workplace outcomes. For instance, employers who proactively address mental health can see significant reductions in absenteeism, presenteeism, and staff turnover.^1^ It can also result in high turnover rates, reduced creativity, and an unfavorable work environment. Organizations that prioritize mental health initiatives see a significant return on investment, with improved employee engagement, morale, and ultimately greater profitability.^2^

 While prioritizing the EMW promises clear benefits, concerns about its cost and implementation remain.^3,4^ This can lead to a “band-aid” approach to the EMW, heavily reliant on an organization’s current financial state.^4,5^

 Adding complexity to the issue are recent social and economic shifts. The COVID-19 pandemic, for instance, blurred the lines between work and home life, impacting employee well-being, particularly for remote and part-time workers. Economic instability, further fueled by the pandemic, injects additional financial stress into organizations, making the EMW investments feel less essential.^6,7^

 However, this is not all. Numerous studies have shown that mental wellbeing in the workplace can be provided to a satisfactory level by implementing some simple and low-cost programs. Of course, this requires targeted, multifaceted, and evidence-based interventions.^8^ For this reason, the main challenge in organizations for providing the EMW is the design of effective interventions to control and improve it.

 While the New Zealand Mental Health Association’s “five ways to mental wellbeing in the workplace” framework^9^ offers valuable predictors for organizational managers to design effective interventions, a key question remains: how effectively can these factors be implemented together without disrupting employees’ daily workflow?

 Employees devote more than eight hours each day to their job responsibilities, often while managing personal matters. Adding on further processes labeled as “wellbeing interventions” may not only fail to improve their mental well-being, but potentially create an additional burden, ultimately achieving the opposite of the intended outcome.^10^ Therefore, interventions should be designed in such a way that they are not time-consuming, do not impose more duties on the employee, are accessible to increase participation, are not costly, and the employee can follow the intervention process with the least possible cost.^11^ Compared to the cost of providing optimal physical equipment and financial benefits for the workforce, these interventions should be less costly and have more impact on the employees.^11^

 The virtual space can provide a desirable platform for intervention designers. Because access to the virtual space via smart phones is available for almost all employees. Monitoring and impacting intervention programs will also be easier for managers, and it will also create less cost than other interventional methods.^8^

 For improving the EMW, various examples have been used in different countries around the world. The “Welbot” is one example of these applications which based on creating a social and interactive network among employees, strengthens organizational relationships and improves mental wellbeing in the workplace.^12^ In Germany, the “ONYA” application has also been designed and developed to improve the mental wellbeing of employees outside of working hours, which provides training based on a five-dimensional pattern.^13^ There are various web-based programs and smart phone applications designed to improve mental well-being that are designed and developed to suit the needs of their audiences and users.^14,15^

 The research team has not found any dedicated application for the EMW in Iran. Additionally, there is no targeted intervention design available in the form of a dedicated application in Iran, based on the five predictors of mental wellbeing in the workplace. In addition, In Iran, international applications are limited or impossible due to cultural, climatic, and working conditions.

 This study aimed to create interventions to improve mental well-being in the workplace in virtual spaces based on five main predictors, and to evaluate their effectiveness. The goal is to provide evidence that can be used by researchers to develop a dedicated application for improving the EMW. Therefore, the present study was conducted to determine the effectiveness of an intervention using the LAWE (loveliness and well-being of employees) application for improving the EMW in the workplace that is designed based on five predictors.

## Methods

 This was a quasi-experimental study with pre-post design in two groups: an intervention group (53 employees) and a non-intervention group (57 employees). To assess the impact of the intervention, pre-tests and post-tests were administered to participants in both the intervention and non-intervention groups.

###  Study participants

 The study was conducted at the Kerman University of Medical Sciences in southeast Iran. Participants were recruited through a voluntary enrollment process. Completing the virtual forms was considered as providing informed consent to participate. Employees without Android smartphones or facing imminent transfers were excluded from the study. Removing identifying information from questionnaires and assigning participant IDs was implemented to ensure participant anonymity and data confidentiality. All data were stored securely with access restricted to the research team.

 The sample size was determined considering practical limitations, such as the availability of eligible participants and resources. Despite these constraints, and based on previous research in this area, we believed the sample size was adequate to detect a significantly effect.

 To ensure adequate sample size and representativeness, four schools were randomly allocated to either the intervention or non-intervention group. Subsequently, a census was conducted among all eligible employees within each assigned school, given the study’s nature and the need for employee interaction. To ensure homogeneity in group size and to prevent contamination bias, the intervention group comprised 53 employees from the School of Public Health and the School of Health Services Management and Information. Due to logistical constraints and to maintain comparability, the non-intervention group was formed by 57 employees from the School of Nursing and Midwifery and the School of Pharmacy. This decision was based on the availability of accessible and cooperative departments. By combining random allocation of schools with a census of eligible employees within each school, we aimed to strengthen the study’s generalizability while maintaining the necessary sample size for rigorous analysis. Also, to ensure group homogeneity, strengthen the generalizability of the findings and ensure that observed differences were due to the intervention rather than baseline characteristics, an analysis of covariance (ANCOVA) was employed.

###  Theoretical principles of interventions

 The interventions were developed based on five predictors of employee mental well-being: “Keep learning”, “Connect”, “Take notice”, “Be active”, and “Given”.^9^

*“Keep learning”* refers to the idea that individuals need to continue learning in their field of work and interests, as there is always something new to discover. This can help to keep their mind and spirit fresh and active. *“Be active”* refers to regular and daily exercise, even at the desk. This can help to ensure physical health and reduce pain caused by insufficient posture. It can also contribute to the individual’s mental well-being. *“Take notice”* refers to the individual’s curious mind. They should be sensitive to the issues and problems in the workplace and seek suitable solutions to address them. They should also be mindful of the small details in the workplace, such as the birthdays of close colleagues, and celebrate them with a simple greeting to create a lively and vibrant work environment. *“Given”* refers to simple and accessible tasks to help others, even caring for plants and animals. Simply being kind and compassionate to the environment around them to the best of their ability throughout the day is adequate to strengthen this factor. *“Connect”* refers to establishing positive, constructive, and sustainable relationships in the workplace among colleagues and other management levels in the organization. Paying attention to this factor can have a powerful impact on job satisfaction, administrative vitality, improving collaboration between departments, and reducing inequalities in an organization.^9^

###  Intervention design

 Before the start of the intervention schedule, an orientation workshop was arranged for the intervention group in collaboration with the faculty heads. The workshop provided an overview of the study’s nature, intervention objectives, and existing ambiguities. Additionally, addressed participant data privacy concerns, explaining how anonymity and confidentiality would be maintained throughout the study such as removing identifying information from questionnaires, assigning participant IDs and secure data storage.

**Figure 1:**
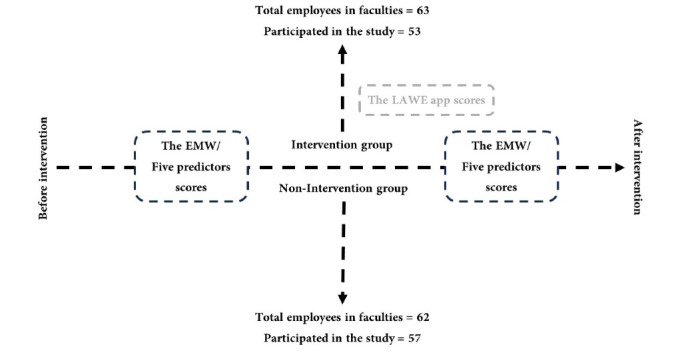
Study design flowchart

 Then, the pre-test questionnaires were given to both the intervention and non-intervention groups. A week after collecting the pre-test information, the intervention started by sending the LAWE app link to install for the intervention group. The intervention duration was 31 days, and at the end of this period, a post-test was conducted in two groups (Figure 1).

###  Overview of the LAWE app

 The interventions were implemented in a virtual environment through an Android smartphone app called the LAWE (Liveliness and Wellbeing of Employees). The main structure of the LAWE app was designed based on five predictors of mental wellbeing in the workplace:

 “Keep learning”: Educational reminders and learning massages were sent daily by LAWE admin on topics such as stress management, mindfulness, effective communication in the workplace, and work-life balance.

 “Be active”: A daily exercise pattern based on 15 standards behind the desk exercises were executed daily for the user.

 “Take notice”: Four important challenges based on issues in the workplace were placed in the app during the intervention period (one challenge per week). Users could provide feedback and solutions to these challenges. The best solutions were awarded special points.

 “Connect”: The possibility of group chat and sending posts and reels on various workplace issues was provided among colleagues. At the end of each week, users could evaluate each other based on their weekly interactions and give each other points.

 “Given”: A list of 50 suggested activities that create a positive feeling in oneself or others was made available to the user. Users could earn points for doing each of these activities.

 In the LAWE app, participants perform gamification tasks and activities to earn points. Beyond the five predictors, additional features were incorporated into the LAWE app to encourage participation. These included an online leaderboard, an offline counselling entitle: the current mood, and the golden post, through which users could earn additional points. All content used in the LAWE app, including learning messages, the daily exercise pattern, the current mood counseling messages, and daily motivational reminders, was derived from similar articles and apps to ensure that users were provided with validated content.

###  Data collection tool

 The current study employed six primary variables, the EMW and five predictors of it, as outlined within the Theoretical Principles of Interventions. EMW levels were assessed using the Persian Version of the Warwick-Edinburgh Mental Wellbeing Scale (Pr-WEMWBS) questionnaire and to measure the other five predictors, five standardized instruments were utilized, as explained below:

*Pr -WEMWBS*:In the present study, the main variable was the EMW. To assess it, Pr-WEMWBS was used. The original Warwick-Edinburgh Mental Wellbeing Scale (WEMWBS) was developed in 2007 and it has demonstrated excellent psychometric properties in previous research, including strong internal consistency (Cronbach’s alpha = 0.91), good construct validity, and satisfactory test-retest reliability,^16^ stands out as a valuable tool for assessing and promoting mental well-being due to its comprehensive, multidimensional, positive, validated, culturally sensitive, easy-to-use, cost-effective, versatile, change-sensitive, and intervention-informing nature. Its widespread adoption and positive feedback from users further solidify its position as a trusted resource in the field of mental well-being. The psychometric properties of Pr-WEMWBS (including validity, reliability, exploratory factor analysis, and confirmatory factor analysis) were fully evaluated in Persian by the research team of the present study. The results of this evaluation published entitled: “Psychometrics Properties of Persian Version of The Warwick-Edinburgh Mental Wellbeing Scale (Pr-WEMWBS)”.^17^ The scale consists of 14 items. Respondents are asked to rate how often they have experienced each item on a scale of 1 (never) to 5 (always). The overall the EMW score is calculated from the sum of the scores, with a higher score indicating better mental wellbeing.

*The Behavioral Regulation in Exercise Questionnaire (BREQ)*: the BREQ is an 18-item questionnaire designed to assess the motivation for exercise. Be Active refers to some measure of physical activity level or participation in exercise and the interventions in this study were designed to enhance employee participation and encourage them to engage in physical activity at work. Therefore, the BREQ could serve as a suitable tool to measure the effectiveness of these interventions. Additionally, the Persian version of this instrument has undergone rigorous psychometric evaluation and has been widely used. The BREQ questionnaire was developed by Markland and Tobin in 2004^18^ and its Persian version was also psychometrically evaluated by Farmanbar and colleagues.^19^ The BREQ has four domains including External regulation, Introjected regulation, Identified regulation, Amotivation. Each item on the BREQ is rated on a five-point Likert scale, with zero indicating strongly disagree and four indicating strongly agree. The total score for the questionnaire is calculated by summing the scores for all items. A higher score indicates higher motivation for exercise.

*The Informal Workplace Learning Questionnaire (IWL)*: the IWL is a 12-item questionnaire designed to assess informal learning in the workplace. Keep learning stems from individuals’ inherent curiosity and desire to acquire new knowledge, which in turn fosters a sense of vitality and rejuvenation. It also encompasses an understanding of the obstacles and benefits that lie along this path. Similarly, the IWL questionnaire aims to gauge the extent to which employees within an organization exhibit an inclination towards learning new things. Therefore, this questionnaire could serve as an appropriate tool for assessing keep learning. Additionally, a standardized Persian version of that was available.

 The questionnaire was developed by Lohman in 2006^20,21^ and the psychometric properties of the Persian version were also evaluated in a study by Momivand in 2013.^22^ The IWL has three domains: Learning activities, Environmental inhibitors, Personal characteristics. Items in the learning activities domain are rated on a 5-point Likert scale, with 0 indicating never and 4 indicating always. Items in the environmental inhibitors and personal characteristics domains are rated on a 5-point Likert scale, with 0 indicating very low and 4 indicating very high. Two additional questions are included at the end of the questionnaire, one for participants to provide suggestions for improving informal learning in the workplace and one for participants to provide demographic information.

*Willingness to Communicate Scale (WTC)*: the WTC is a 12-item questionnaire designed to estimate the connect level by assessing willingness to communicate. The questionnaire was developed by James and McCroskey in 1992 and was widely published in 2009.^23^ The connect aims to assess the extent and intensity of work-related and personal interactions among colleagues within an organization. It delves into the level of positive communication that exists among employees. The first step to identifying the level of this predictor is to understand the range to which an employee is inclined to engage in communication. Therefore, the WTC serves as a valuable tool for this purpose. Additionally, a standardized Persian version of this instrument is readily available.

 The Persian version of the questionnaire was used in a study by Bayrami and colleagues in 2012, and it was found to have good validity and reliability.^24^ The WTC has two subscales: Communication Apprehension and Communication Confidence. Each item on the WTC is rated as either “yes” or “no.” The total score for the questionnaire is calculated by summing the scores for all items. A higher score indicates a higher willingness to communicate.

*Curiosity and Exploration Inventory (CEI)*: The CEI is a 7-item questionnaire designed to assess the level of curiosity in individuals was used to assess the take notice level. The take notice or Attention to detail refers to the extent to which an employee pays close attention to the intricacies of their work and finds value in minor details. This trait often manifests in individuals with a heightened sense of curiosity and a problem-solving mindset. Assessing an individual’s level of curiosity can significantly aid in evaluating this predictor. Therefore, the CEI serves as a suitable tool for measuring this predictor. Additionally, a psychometrically validated Persian version of this instrument is readily available. The questionnaire was developed by Kashdan and colleagues in 2004^25^ and the Persian version was psychometrically evaluated by Kaveh Farsani and colleagues in 2015.^26^

 The CEI has two subscales: Exploration and Absorption. Each item on the CEI is rated on a seven-point Likert scale, with 1 indicating strongly disagree and 7 indicating strongly agree. The total score for the questionnaire is calculated by summing the scores for all items. A higher score indicates a higher level of curiosity.

*Emotional Empathy Scale (EES)*: the EES is a 33-item questionnaire designed to assess the level of emotional empathy and using for assessing the given level. The Given embodies kindness and compassion, not just towards fellow humans but towards all living beings, including animals, plants, and the environment. The EES serves as a well-established and widely recognized tool for measuring individuals’ levels of kindness. Its Persian version has been extensively used for many years. The questionnaire was developed by Mehrabian and Epstein in 1972 and the Persian version was psychometrically evaluated by Zarshghaee and colleagues in 2009.^27^ The EES has seven subscales: Reactive empathy, Expressive empathy, Participatory empathy, Emotional reactivity, Emotional stability, Empathy for others, Emotional control. Each item on the EES is rated on a five-point Likert scale, with 1 indicating strongly disagree and 5 indicating strongly agree. The total score for the questionnaire is calculated by summing the scores for all items. A higher score indicates a higher level of emotional empathy.

###  Statistical analysis

 Mean and standard deviation were used to describe the average scores of the study variables. Paired-samples t-test was employed to assess the pre- and post-intervention differences in scores. The ANCOVA was employed to evaluate the effectiveness of the intervention and determine normality, variance homogeneity and linearity of data. Independent-samples t-test and one-way ANOVA were used to compare the mean scores of the variables across the intervention group and explore the effects of demographic variables on the outcome measure among the intervention group. Pearson’s correlation coefficient was calculated to examine the relationship between predictors and the EMW. All analyses were conducted using IBM SPSS Statistics, version 28 with a significance level of 0.05 to determine the effectiveness of the intervention. To further mitigate the influence of confounding variables, the current study employed two additional strategies for data and response validation. Firstly, the scores obtained from the questionnaires were compared with the points earned within the application for each domain, which were accessible to the intervention group. This served as a comparative path to verify the accuracy of the data. Secondly, the usage time of the LAWE by each user and its impact on their EMW level were examined as another means of validating the collected data.

 To ensure data completeness, the questionnaire was designed with a forced-choice format, requiring participants to provide an answer for each question. This approach eliminated missing data entirely, resulting in a robust and reliable dataset.

 To minimize the potential impact of confounding variables, a rigorous methodological approach was adopted. A detailed explanation of how potential confounding variables were controlled for can be found in the *study bias*.

## Results

 After screening for inclusion and exclusion criteria, 53 of the 63 employees in the intervention group faculties were enrolled in the study. Similarly, 57 of the 62 eligible employees in the non-intervention group faculties completed both pre- and post-intervention questionnaires (Table 1).

**Table 1:** Demographic variables of the participants in both groups

**Characteristics**	**Total (n=110)**	**Intervention group (n=53)**	**Non-intervention group (n=57)**	***P*** ** value**
Age (y)	< 25 (%)	5 (4.5)	2 (3.8)	3 (5.3)	< 0.01
	25 to 35 (%)	33 (30.0)	7 (13.2)	26 (45.6)	
	35 to 45 (%)	46 (41.8)	28 (52.8)	18 (31.6)	
	45 to 55 (%)	22(20.0)	14 (26.4)	8 (14.0)	
	> 55(%)	4 (3.6)	2 (3.8)	2 (3.5)	
Gender	Female (%)	89 (80.9)	42 (79.2)	47 (82.5)	0.67
	Male (%)	21 (19.1)	11 (20.8)	10 (17.5)	
Mariage status	Married (%)	101 (91.8)	49 (92.5)	52 (91.2)	0.81
	Bachelor/ single (%)	9 (8.2)	4 (7.5)	5 (8.8)	
Job position	Staff (%)	64 (58.2)	25 (47.2)	39 (68.4)	0.06
	Expert (%)	45 (40.9)	27 (50.9)	18 (31.6)	
	Manager/administrator (%)	1 (0.9)	1 (1.9)	0 (0.0)	
Educational level	BA (%)	75 (68.2)	37 (69.8)	38 (66.7)	0.72
	MA (%)	35 (31.8)	16 (30.2)	19 (33.3)	
	PhD (%)	0 (0.0)	0 (0.0)	0 (0.0)	
Income level	< Commune average (%)	17 (15.5)	12 (22.6)	5 (8.8)	0.06
	= Commune average (%)	91 (82.7)	40 (75.5)	51 (89.5)	
	> Commune average (%)	2 (1.8)	1 (1.9)	1 (1.8)	
Number of children	None (%)	5 (4.5)	0 (0.0)	5 (8.8)	< 0.01
	One (%)	12 (10.9)	5 (9.4)	7 (12.3)	
	Two (%)	28 (25.5)	6 (11.3)	22 (38.6)	
	≥ Three (%)	65 (59.1)	42 (79.2)	23 (40.4)	
Work history (year)	< Five (%)	8 (7.3)	2 (3.8)	6 (10.5)	< 0.01
	Five to 10 (%)	17 (15.5)	4 (7.5)	13 (22.8)	
	10 to 15 (%)	42 (38.2)	16 (30.2)	26 (45.6)	
	> 15 (%)	43 (39.1)	31 (58.5)	12 (21.1)	

 ANCOVA revealed that the assumption of homogeneity of variances was met (F = 2.34,* P* = 0.122). The post-intervention mean score was significantly higher in the intervention group compared to the non-intervention group by 0.219 points (*P* < 0.01). The calculated regression coefficient was 0.39, indicating that the linear model accounted for 39% of the variance in the primary outcome variable, psychological well-being, within the intervention group (Table 2).

**Table 2:** Deference means scores and ANCOVA test results between two group

**Variable**s	**Deference between both groups**			
	**Pre-test**		**Post-test**	
	***t***	***P *** **value**	***t***	***P *** **value**
MWE	1.36	0.17	5.69	< 0.01
Be active	0.69	0.49	0.85	0.39
Keep learning	-1.00	0.31	2.52	< 0.05
Take notice	0.89	0.37	1.07	0.28
Given	1.60	0.11	2.98	< 0.01
Connect	-1.86	0.06	2.68	< 0.01
**ANCOVA test of between-subjects effects**				
**Groups**	***P *** **value**	**Partial Eta squared**	**R squared**	**Adjusted R squared**
Covariant (Pre-test)	< 0.001	0.370	0.399	0.388
Intervention/ non-intervention	< 0.001	0.219		

 Also, before the intervention, there was no significant difference between the mean scores of the EMW between the intervention and non-intervention groups (*P* = 0.17). However, after the intervention, this difference became significant (*P* < 0.01). In addition, before the intervention, there was no significant difference in the mean scores of any of the predictors between the intervention and non-intervention groups; however, after the intervention, the mean scores in the “Keep learning” *(P* < 0.05), the “Given” (*P* < 0.01), and the “Connect” (*P* < 0.01) showed a significant difference. However, still in the “Be active” (*P* = 0.39) and the “Take notice” (*P* = 0.28) no significant difference was observed (Table 2).

 In the intervention group, the difference between the mean scores of the EMW before the intervention (52.26 ± 8.61) and after the intervention (60.01 ± 6.95) was significant (*P* < 0.01). In addition, the difference between the mean scores of all predictors except the “Take notice” (*P* = 0.17) before and after the intervention was significant. However, in the non-intervention group, the mean scores before and after in any of the variables showed a significant difference (Table 3).

**Table 3:** Mean scores before and after the intervention in both groups

**Variable**s	**Intervention group (n=53)**			**Non-intervention group (n=57)**		
	**Pre-test**	**Post-test**	***P *** **value**	**Pre-test**	**Post-test**	***P *** **value**
	**Mean±SD**	**Mean±SD**		**Mean±SD**	**Mean±SD**	
MWE	52.26 ± 8.61	60.01 ± 6.95	< 0.01	50.07 ± 8.21	51.40 ± 8.74	0.43
Be active	40.39 ± 8.03	43.45 ± 8.08	< 0.01	39.12 ± 10.94	42.00 ± 9.62	0.11
Keep learning	3.10 ± 0.55	3.65 ± 0.48	< 0.01	3.20 ± 0.46	3.39 ± 0.55	0.07
Take notice	35.07 ± 7.02	36.33 ± 6.25	0.17	33.71 ± 8.71	34.87 ± 7.87	0.55
Given	112.0 ± 13.32	118.0 ± 16.13	< 0.01	107.7 ± 14.24	110.2 ± 14.05	0.06
Connect	7.18 ± 3.91	9.66 ± 2.28	< 0.01	8.45 ± 3.20	8.15 ± 3.42	0.29

 In the intervention group, the mean score of the EMW before and after the intervention showed a significant difference only based on age. This difference was not significant based on other demographic variables of the participants. In the non-intervention group, the mean score of the EMW did not show a significant difference based on any of the background variables (Table 4).

**Table 4:** Mean scores of EMW before/ after the intervention in both groups regarding to characteristics

**Characteristic**s		**Total mean of EMW **					
		**Intervention (n=53)**			**Non-intervention (n=57)**		
		**Mean±SD**		***P *** **value**	**Mean±SD**		***P *** **value**
**Pre-test**	**Post-test**	**Pre-test**	**Post-test**				
Age (y)	< 25	54.00 ± 1.41	55.00 ± 4.24	< 0.05	38.67 ± 4.16	41.33 ± 9.07	0.11
	25 to 35	47.86 ± 7.34	62.00 ± 5.66		47.46 ± 6.21	48.62 ± 7.41	
	35 to 45	52.89 ± 9.47	61.36 ± 6.47		51.72 ± 8.98	52.44 ± 8.58	
	45 to 55	52.21 ± 8.24	57.14 ± 7.59		55.38 ± 4.21	61.63 ± 3.78	
	> 55	57.50 ± 4.95	59.50 ± 13.44		65.00 ± 1.41	52.50 ± 4.95	
Gender	Female	52.17 ± 8.49	60.24 ± 7.13	0.56	50.30 ± 8.28	50.62 ± 8.89	0.06
	Male	52.64 ± 9.49	59.18 ± 6.51		49.00 ± 8.27	55.10 ± 7.28	
Mariage status	Married	52.63 ± 8.60	60.65 ± 6.65	0.38	50.62 ± 8.19	51.92 ± 8.62	0.9
	Bachelor/ single	47.75 ± 8.54	52.25 ± 6.64		44.40 ± 6.88	46.00 ± 9.08	
Job position	Staff	52.72 ± 7.50	60.24 ± 6.92	0.35	49.90 ± 7.83	50.87 ± 8.91	0.66
	Expert	51.70 ± 9.77	60.07 ± 7.11		50.44 ± 9.23	52.56 ± 8.49	
	Manager/administrator	56.00 ± 0	53.00 ± 0		0 (0.0)		
Educational level	BA	50.95 ± 8.38	59.11 ± 7.38	0.56	47.84 ± 7.94	50.50 ± 8.78	0.11
	MA	55.31 ± 8.65	62.13 ± 5.49		54.53 ± 7.01	53.21 ± 8.60	
	PhD	0 (0.0)	0 (0.0)				
Income level	< Commune average	49.25 ± 8.04	57.17 ± 5.46	0.99	45.20 ± 7.73	47.80 ± 11.03	0.52
	= Commune average	53.40 ± 8.63	61.10 ± 7.09		50.67 ± 8.21	51.69 ± 8.61	
	> Commune average	43.00 ± 0	51.00 ± 0		44.00 ± 0	55.00 ± 0	
Number of children	None	0 (0.0)	0.41	44.40 ± 6.88	46.00 ± 9.08	0.99
	1	49.20 ± 8.07		53.40 ± 6.31	41.14 ± 9.62		42.86 ± 11.08
	2	51.83 ± 11.50		62.33 ± 4.72	47.23 ± 5.25		48.86 ± 6.71
	≥ 3	52.69 ± 8.39		60.48 ± 6.96	56.74 ± 4.96		57.61 ± 5.14
Work history (year)	< 5	54.00 ± 1.41	55.00 ± 4.24	0.57	43.17 ± 6.40	46.33 ± 8.16	0.77
	5 to 10	47.00 ± 8.41	63.50 ± 3.87		46.54 ± 5.49	49.46 ± 7.69	
	10 to 15	51.88 ± 8.79	60.37 ± 6.56		50.12 ± 8.43	50.19 ± 9.00	
	> 15	53.03 ± 8.85	59.71 ± 7.53		57.25 ± 5.83	58.67 ± 5.52	

 In the intervention group, mean scores of the EMW after the intervention was only significantly correlated with the “Connect” (*P* < 0.01). Although the other predictors improved after the intervention, mean scores of them didn’t show a significant correlation with the mean scores of the EMW after the intervention. However, the “Keep learning” and the “Given” showed a significant correlation with each other (*P* < 0.05). the “Be active” also showed a significant correlation with the two predictors: the “Connect” *(P* < 0.05) and the “Take notice” (*P* < 0.01). On the other hand, the “Connect” was also significantly correlated with the “Take notice”, and the “Take notice” showed a significant correlation with the “Given” (*P* < 0.05) (Table 5).

**Table 5:** Correlations between EMW and predictors post-test scores among the intervention group

**Variables **	**MWE**	**KL**	**BA**	**C**	**TN**	**G**
Means of EME (EMW)	Pearson correlation		-0.012	0.187	0.388	0.252	0.230
	*P* value		0.933	0.181	< 0.01	0.068	0.097
Means of Keep Learning (KL)	Pearson correlation			0.073	0.029	-0.116	-0.488
	*P* value			0.601	0.837	0.406	< 0.05
Means of Be Active (BA)	Pearson correlation				0.346	0.498	0.180
	*P* value				< 0.05	< 0.01	0.122
Means of Connect (C)	Pearson correlation					0.331	0.165
	*P* value					< 0.05	0.198
Means of Take Notice (TN)	Pearson correlation						0.367
	*P* value						< 0.05
Means of Given (G)	Pearson correlation						-
	*P* value						

## Discussion

 In the present study, the difference in the mean score of the EMW between the intervention and non-intervention groups before using the LAWE app was not significant, but after using the LAWE app, it became significant. On the other hand, the use of the LAWE app showed a desirable effectiveness for improving the level of the EMW. To examine the effectiveness of the intervention, an ANCOVA was conducted with the EMW as the outcome variable. This finding suggests that the intervention was effective in improving psychological well-being among participants.

 These findings indicate the effectiveness of using the LAWE application. Digital technologies and virtual spaces are proving to be valuable tools in healthcare, and mental well-being is no exception. Studies have consistently shown their effectiveness. Armaou and colleagues’ review highlights the significant impact of digital tools across the field.^8^ van der Feltz-Cornelis et al demonstrated how their EMPOWER software not only boosted workplace mental well-being but also reduced stress and increased employee satisfaction. The benefits of digital tools extend beyond intervention design.^28^ Sierk et al leveraged them to assess workplace health and well-being.^29^ Similarly, Woodward et al explored the potential of mobile apps in this area, emphasizing their growing significance in promoting workplace well-being.^15^ Additionally, empowering individuals in this area is crucial. Mastering digital skills, particularly using smart applications, has become an essential competence for modern employees.^15^ To facilitate this, meticulous attention should be paid to designing applications based on precise, targeted, and evidence-based principles. Karlsen et al emphasized this point in their review, finding that nearly all dedicated apps for promoting mental well-being and occupational health were built upon rigorous research foundations.^30^

 Another important point to note in this study is that the LAWE app has a comprehensive, multifaceted, and specialized design. Detailed information about the LAWE app is provided by the research team in a study titled “LAWE app: An Appropriate Platform for Promoting EMW” that is currently under review and publication. Many of the existing apps focus on one or two aspects of employee well-being or are specifically designed for a specific audience, such as working women. For example, in the study by Coelhoso et al, the app was designed to reduce job stress among working women.^31^ Collins et al evaluated a specialized app for reducing depression in the workplace, which would indirectly affect the promotion of employee well-being.^32^ Unlike many of these apps, the LAWE app is designed with an evidence-based, comprehensive, and multifaceted approach and specifically aims to promote the EMW by impacting the main predictors in this area.

 It’s crucial to note that all predictor scores increased after using the LAWE app compared to before. This clearly demonstrates the LAWE’s direct influence on the factors that promote the EMW. While the changes for “Take notice” and “Be active” were not statistically significant, they still showed positive growth following the intervention. This result for “Be active” likely stems from the existing emphasis on physical activity within most organizations. Regular exercise programs, both individual and group sessions, are often commonplace, leading to a relatively consistent level of “Be active” across employee groups. Additionally, many organizations prioritize employee well-being and have adopted dedicated physical activity apps, especially for desk-bound staff.^33,34^ This existing focus potentially minimizes the need for further “Be active” promotion within the EMW interventions. Overall, the LAWE’s effectiveness is evident in raising the levels of all key predictors of the EMW, demonstrating its potential as a valuable tool for improving employee well-being.

 The difference observed between the intervention and non-intervention groups was also not significant about “Take notice”. On the one hand, given the development of communication and social networks between colleagues, it was possible that the intervention group employees would inform other colleagues about the nature of the study and the attractive features of the LAWE app.^35^ Therefore, it was predictable that curiosity among other employees in the organization would increase compared to the intervention group, and this issue would indirectly affect “Take notice”. This issue could be a reason for the non-significance of the difference in its score in the intervention and non-intervention groups. In addition, employees working in an organization naturally have a high level of curiosity and attention to new programs and interventions, and this issue also causes employees to show a high level of curiosity and attention in almost all items.^35,36^

 While the intervention group showed positive changes in three other predictors, the difference in “Take notice” scores between the intervention and non-intervention groups wasn’t statistically significant. This may be due to several factors. First, the close-knit nature of workplaces may have led to spillover effects. Employees in the intervention group likely shared their experiences with the LAWE app and the study with colleagues, potentially sparking curiosity and indirect engagement among those in the non-intervention group. This shared interest could have boosted “Take notice” scores across both groups, masking the direct impact of the intervention. Second, a general organizational curiosity towards new initiatives is also a likely factor. Employees are naturally keen on innovative programs and interventions, potentially leading to an overall heightened awareness regardless of direct participation. This heightened baseline could have made it harder to detect a significant difference in “Take notice” between the two groups. Overall, while the lack of significant difference in “Take notice” requires further investigation, the positive changes observed in other predictors are promising. Future studies with stricter control measures may be needed to fully understand the specific impact of the LAWE app on “Take notice” and further refine its effectiveness.

 Beyond the direct influence of each factor on the EMW scores, the study also revealed significant “Connections between certain factors themselves. These connections can indirectly impact the EMW. For example, the Spearman correlation test showed a link between “Keep learning” and “Given” scores. This suggests that increased employee awareness and knowledge in the workplace can play a key role in fostering kindness among colleagues. The term “Given” in this study encompasses concepts like forgiveness and generosity towards others. Cornish et al. highlight that increasing knowledge can be an effective intervention strategy for individuals experiencing setbacks, by helping them develop self-forgiveness skills.^37^ And knowledge as a crucial element in strengthening the trait of forgiveness. Interestingly, the LAWE app’s “Given” section was designed to nudge users towards these desired behaviours by offering a pre-designed list of 50 specific activities. This essentially provided a form of daily, subtle education, simultaneously reinforcing both “Keep learning” and “Given” processes.

 The “Be active,” “Connect,” and “Take notice” factors showed a surprising intertwining. Research by Jindo et al confirms the positive impact of regular workplace exercise on employee relations, job satisfaction, and collaboration.^38^ This aligns with interesting observations within our study. Firstly, organizational culture itself often fosters shared exercise routines. Many employees participate in morning exercise programs together, or engage in quick desk stretches or walks with colleagues. In fact, solo workouts are less common; group exercise seems to be the norm, especially within departments. This creates a built-in opportunity for regular interaction and effective communication.^39^ Secondly, Lievens et al suggest that paying attention to details and practicing curiosity in the workplace are key ingredients for strong bonds between colleagues.^35^ This ties closely to the “Take notice” factor. The enhanced communication stemming from group exercise likely amplifies these qualities, further strengthening connections and mutual understanding. Therefore, it appears that promoting physical activity in the workplace not only directly benefits individual well-being but also indirectly fosters a culture of connection and “Take notice” ultimately contributing to a more supportive and collaborative work environment.

###  Study biases

 There were several potential biases that could had affected the results. For Selection Bias both groups were defined in such a way that all employees working in the administrative departments of the target faculties were included to ensure that they had similar job descriptions and were in an equal position based on conditions and motivation. Additionally, the faculties in the intervention group were selected because the research team had a history of collaboration with these faculties and could better contribute to the regular implementation of the intervention program. To address history bias, since both groups consisted of employees from the same university, and any probable programs implemented outside of the intervention program were consider for all employees during the intervention period. This should minimize history bias between the two groups. However, the present study was designed using a quasi-experimental approach, as it was not possible to control all external confounders for the intervention and control groups. For reduction Maturation bias, the pre-posttest approach was considered in the study. This would have allowed you to compare the change in mental well-being from pre-test to post-test between the two groups, which would help to control for any natural changes that occurred over time. To mitigate contamination bias, participants from the intervention and control groups were assigned to separate faculties to minimize their interactions and exposures during the intervention period. Additionally, the intervention program and its accompanying activities were delivered through the LAWE. Participants in the intervention group received unique confidential codes to access the app.

## Conclusion

 The LAWE app provides a desirable and comprehensive platform for improving the EMW in organizations. It can significantly improve most of the main EMW predictors. So, it is a valuable resource for organizations looking to improve the well-being of their employees. Additionally, some characteristics such as age, can also affect the use of the LAWE app and, consequently, the effectiveness of interventions based on it. For example, older employees may be less likely to use the app or may not find it as engaging as younger employees. This is because older employees may be less familiar with technology or may have different preferences. Organizations that are considering using the LAWE app can provide training and support to employees on how to use the app. By taking these steps, organizations can maximize the effectiveness of the LAWE app and improve the well-being of employees of all ages.

## Implications for practice

 The primary users of this application are employees and managers within organizations. Employees can utilize its daily tasks and features to enhance their mental wellbeing and foster a supportive workplace culture. Managers can leverage the application to create a healthier work environment, reduce burnout, and make evidence-based decisions regarding talent management. To maximize the application’s impact, organizations may consider integrating it into existing wellness programs, providing comprehensive training for employees and managers, and developing policies that support mental health and well-being. Challenges such as resistance to change and privacy concerns should be proactively addressed through effective communication and data security measures.

## Study Limitations

 The current version of the LAWE app is limited to Persian, which may restrict its applicability to non-Persian speaking populations. However, efforts are underway to develop English and potentially other language versions. Some organizations may have policies restricting the sharing of work-related content on social media platforms, which could potentially hinder the use of LAWE. Implementing a dedicated server for organizational use could mitigate this issue. The presence of numerous uncontrolled confounding variables limits the ability to establish definitive causal relationships. Replicating the study in similar populations and employing rigorous data validation methods were used to address this limitation. Future research should explore the impact of these variables in more depth. The initial development of LAWE for the Android platform restricted its accessibility. Expanding the app’s reach to Windows and iOS platforms is a priority for future development. Future research should focus on evaluating the long-term effects of LAWE on employee well-being, exploring its effectiveness in different organizational contexts, and investigating the potential for integrating LAWE with other organizational wellness initiatives. Additionally, studies examining the cost-benefit analysis of implementing LAWE would be valuable.

## Acknowledgments

 We would like to acknowledge the Vice-Chancellery for Research & Technology at Shahid Sadoughi University of Medical Sciences in Yazd Province. Also, we would like to acknowledge Dr. Seyed Vahid Ahmadi Tabatabaei, assistant Professor of health education and health promotion in the Department of Health Education and Promotion at Kerman University of Medical Sciences.

## Competing Interests

 The authors declare that they have no conflict of interest.

## Data Availability Statement

 The data underlying the findings of this study are readily available from the corresponding author upon reasonable request.

## Ethical Approval

 All authors are committed to entering, reviewing, analyzing and reporting all the data and documents without bias and minimal interference. Also, its confirmed that the research meets ethical guidelines and adheres to the legal requirements of the study country. The ethical code number IR.SSU.SPH.REC.1402.031 from Shahid Sadoughi University of Medical Sciences confirms all aspects of this study. Furthermore, filling out the forms in the virtual system was voluntary and was considered as giving informed consent to participate in the study.
